# Systemic immunity markers associated with lymphocytes predict the survival benefit from paclitaxel plus bevacizumab in HER2 negative advanced breast cancer

**DOI:** 10.1038/s41598-021-85948-2

**Published:** 2021-03-18

**Authors:** Shogo Nakamoto, Masahiko Ikeda, Shinichiro Kubo, Mari Yamamoto, Tetsumasa Yamashita, Akifumi Notsu

**Affiliations:** 1 Division of Breast and Thyroid Gland Surgery, Fukuyama City Hospital, 5-23-1 Zao, Fukuyama, Hiroshima Japan; 2 Division of Clinical Research Center, Shizuoka Cancer Center, Shizuoka, Japan

**Keywords:** Breast cancer, Medical research, Biomarkers, Oncology, Cancer therapy, Chemotherapy

## Abstract

Although paclitaxel plus bevacizumab (PB) therapy is an effective chemotherapeutic regimen for HER2-negative advanced breast cancer (ABC), predictive markers for its effectiveness remain undefined. We investigated the usefulness of systemic immunity markers associated with lymphocytes as predictive markers for PB therapy in patients with HER2-negative ABC. We retrospectively reviewed data from 114 patients with HER2-negative ABC who underwent PB therapy from November 2011 to December 2019. We calculated the absolute lymphocyte count (ALC), neutrophil-to-lymphocyte ratio (NLR), platelet-to-lymphocyte ratio (PLR), and lymphocyte-to-monocyte ratio (LMR) as representative systemic immunity markers. The time to treatment failure (TTF) and overall survival (OS) of the patients with high ALC, low NLR, and high LMR were significantly longer compared with those of the patients with low ALC, high NLR, and low LMR. A multivariable analysis revealed that high ALC, low NLR, and low PLR were independent predictors for TTF and high ALC, low NLR, and high LMR were independent predictors for OS. Systemic immunity markers were significantly associated with longer TTF and OS in patients who underwent PB therapy and may represent predictive markers for PB therapy in patients with HER2-negative ABC.

## Introduction

Paclitaxel plus bevacizumab (PB) therapy increases the progression-free survival (PFS) and overall response rate (ORR) of patients with human epidermal growth factor receptor 2 (HER2)-negative advanced breast cancer (ABC)^1–4^. The French Epidemiological Strategy and Medical Economics study reported a significant increase in overall survival (OS) with PB therapy for HER2-negative ABC^5^. In an earlier study, we showed significant increases in the time to treatment failure (TTF) and ORR for HER2-negative ABC and identified a patient subgroup in which OS benefited from PB therapy through propensity score matching^6^. However, biomarkers that identify patients who will experience a survival benefit from PB therapy remain unclear^3,7^.

Inflammatory cells and mediators in the tumor microenvironment play an important role in cancer progression^8^. The presence of an elevated peripheral neutrophil-to-lymphocyte ratio (NLR), a marker of systemic immunity, has been recognized as a poor prognostic factor in various cancers^9,10^. The absolute lymphocyte count (ALC)^11^, platelet-to-lymphocyte ratio (PLR)^12^, and lymphocyte-to-monocyte ratio (LMR)^13^ are also useful systemic immunity markers as a combined prognostic factor. The usefulness of these systemic immunity markers in association with lymphocytes as a combined prognostic marker has been investigated in breast cancer^14–17^. Studies have identified NLR and ALC as predictive markers for eribulin mesylate (eribulin) therapy for ABC^18–20^ and bevacizumab therapy for advanced non-small-cell lung cancer and metastatic colorectal cancer^21,22^. However, it is unclear whether these systemic immunity markers are useful as predictive markers for PB therapy in patients with ABC. Therefore, the aim of this retrospective study was to evaluate the effectiveness of these systemic immunity markers (ALC, NLR, PLR, and LMR) for predicting TTF and OS associated with PB therapy for HER2-negative ABC.

## Results

### Patient characteristics

We enrolled 122 patients with HER2-negative ABC who underwent PB therapy at the Fukuyama City Hospital (Japan) from November 2011 to December 2019. We excluded one patient due to missing systemic immunity marker data and seven patients who had not undergone at least two cycles of PB therapy. The final study sample included 114 patients whose characteristics at the beginning of PB therapy are shown in Table 1. Of these, 82 (71.9%) were estrogen receptor (ER)-positive, 93 (81.6%) had multiple (≥ 3) metastatic sites, and 93 (81.6%) had visceral metastases. Patient characteristics based on systemic immunity marker status are shown in Supplemental Table S1. Patients exhibiting high ALC had previously undergone fewer chemotherapy regimens for ABC compared with those exhibiting low ALC. There was no difference between the high and low NLR groups, high and low PLR groups, or high and low LMR groups (Supplemental Table S1).

**Table 1:** Patient characteristics at the beginning of paclitaxel plus bevacizumab therapy.

Variables	Number of patients (%)
**Age, years, median (range)**	62.0 (32–89)
**ER status**	
Positive	82 (71.9)
Negative	32 (28.1)
**Diagnosis**	
Advanced	44 (38.6)
Recurrence	70 (61.4)
**Metastatic sites**	
CNS	10 (8.8)
Bone	61 (53.5)
Lungs	55 (48.2)
Pleura and/or lymphangiopathy	46 (40.4)
Lymph node	90 (78.9)
Liver	53 (46.5)
Soft tissue	73 (64.0)
**Type of metastases**	
Visceral	93 (81.6)
Non-visceral	21 (18.4)
Number of metastatic sites, median (range)	3.5 (1–8)
**Number of metastatic sites**	
≥ 3	93 (81.6)
< 3	21 (18.4)
Prior (neo) adjuvant chemotherapy*	
Yes	37 (32.5)
No	77 (67.5)
**Disease-free interval**	
< 24 months	67 (58.8)
≥ 24 months	47 (41.2)
**Number of previous chemotherapies**	
0–1	89 (78.1)
≥ 2	25 (21.9)

*CNS* central nervous system, *ER* estrogen receptor.

*Chemotherapy included anthracycline and/or taxane.

### Overall efficacy

The median follow-up time for the study was 17.9 months. The median chemotherapy regimen line before PB therapy was 0 (range 0–10). The overall efficacy of PB therapy for all included patients was as follows: ORR, 69.3%; median TTF, 210 days (95% confidence interval [CI] 196–250); and median OS, 538 days (95% CI 428–703).

### Correlation between systemic immunity markers and TTF

**Figure 1:**
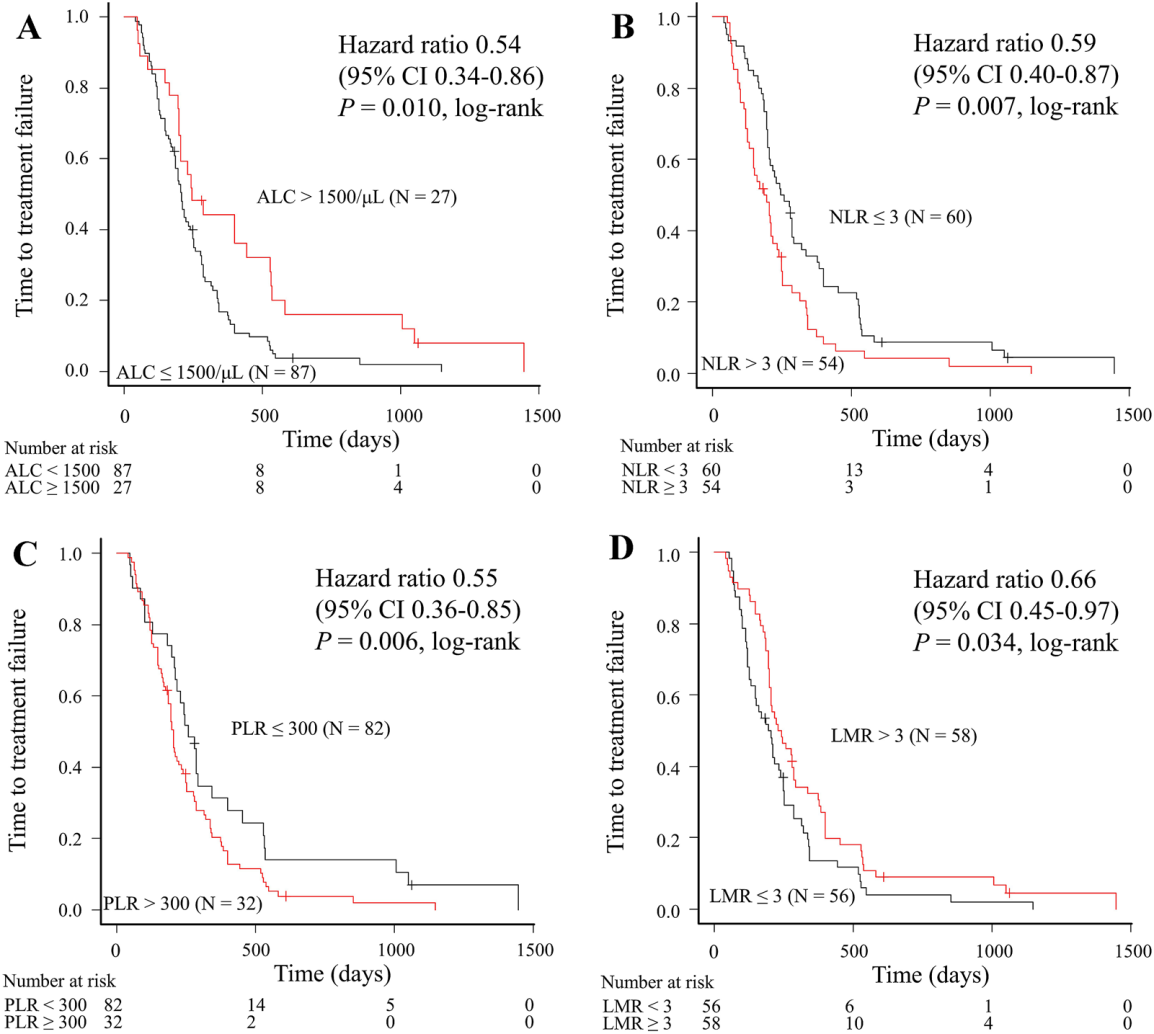
Time to treatment failure according to baseline levels of (**A**) ALC, (**B**) NLR, (**C**) PLR, and (**D**) LMR in patients treated with paclitaxel plus bevacizumab. *ALC* absolute lymphocyte count, *CI* confidence interval, *LMR* lymphocyte-to-monocyte ratio, *NLR* neutrophil-to-lymphocyte ratio, *PLR* platelet-to-lymphocyte ratio.

We compared TTF relative to the systemic immunity markers (Fig. 1). The TTF for patients with high ALC, low NLR, low PLR, and high LMR was significantly longer compared with that for patients with low ALC (245 days vs. 209 days, log-rank *P* = 0.010; Fig. 1A), high NLR (252 days vs .186 days, log-rank *P* = 0.007; Fig. 1B), high PLR (244 days vs. 173 days, log-rank *P* = 0.006; Fig. 1C), and low LMR (236 days vs. 196 days, log-rank *P* = 0.034; Fig. 1D), respectively.

We performed univariable and multivariable analyses to evaluate independent predictors of PB therapy (Tables 2 and 3). Each of the four multivariable analyses identified high ALC, low NLR, and low PLR as independent predictive markers for TTF (*P* = 0.013, *P* = 0.023, and *P* = 0.004, respectively). The respective results of the four multivariate Cox regression analyses are shown in Supplemental Table S2.

**Table 2:** Univariable analysis of time to treatment failure and overall survival (Cox hazard model).

Variables	TTF			OS		
	HR	95% CI	*P*	HR	95% CI	*P*
**Age (≥ 65 vs. < 65 years)**	1.16	0.79–1.70	0.454	1.27	0.81–1.98	0.295
**ER (negative vs. positive)**	1.09	0.72–1.67	0.678	1.02	0.63–1.66	0.929
**Diagnosis (recurrence vs. advanced)**	1.36	0.92–2.00	0.127	1.35	0.86–2.14	0.195
**Metastatic sites (yes vs. no)**						
CNS	1.28	0.66–2.47	0.463	1.39	0.67–2.89	0.380
Bone	0.96	0.65–1.40	0.818	0.98	0.63–1.53	0.945
Lung	0.58	0.39–0.86	0.007	0.65	0.42–1.02	0.060
Pleura and/or lymphangiopathy	0.97	0.66–1.43	0.876	0.83	0.53–1.30	0.425
Lymph node	0.97	0.61–1.54	0.895	0.77	0.46–1.31	0.334
Liver	1.37	0.93–2.00	0.110	1.44	0.92–2.24	0.109
Soft tissue	0.76	0.51–1.14	0.184	0.92	0.59–1.45	0.729
**Visceral metastasis (yes vs. no)**	0.81	0.50–1.31	0.392	0.96	0.55–1.68	0.880
**Number of metastatic sites (≥ 3 vs. < 3)**	0.70	0.43–1.14	0.153	0.79	0.45–1.38	0.403
Prior (neo) adjuvant chemotherapy* (yes vs. no)	1.54	1.02–2.32	0.039	1.28	0.80–2.05	0.304
**Disease-free interval (< 24 months vs. ≥ 24 months)**	1.16	0.79–1.70	0.438	1.23	0.79–1.91	0.367
**Number of previous chemotherapies (< 2 vs. ≥ 2)**	0.90	0.57–1.41	0.644	0.46	0.28–0.75	0.002
**Marker of systemic immunity**						
ALC > 1500/μL versus ALC ≤ 1500/μL	0.54	0.34–0.86	0.010	0.38	0.21–0.71	0.002
NLR ≤ 3 versus NLR > 3	0.59	0.40–0.87	0.007	0.54	0.35–0.84	0.006
PLR ≤ 300 versus PLR > 300	0.55	0.36–0.85	0.006	0.65	0.40–1.06	0.083
LMR > 3 versus LMR ≤ 3	0.66	0.45–0.97	0.034	0.58	0.37–0.90	0.016

*ALC* absolute lymphocyte count, *CI* confidence interval, *CNS* central nervous system, *ER* estrogen receptor, *HR* hazard ratio, *LMR* lymphocyte-to-monocyte ratio, *NLR* neutrophil-to-lymphocyte ratio, *OS* overall survival, *PLR* platelet-to-lymphocyte ratio, *TTF* time to treatment failure.

*Chemotherapy included anthracycline and/or taxane.

**Table 3:** Multivariable analysis of time to treatment failure and overall survival (Cox hazard model).

Variable	TTF			OS		
	HR	95% CI	*P*	HR	95% CI	*P*
ALC > 1500/μL versus ALC ≤ 1500/μL	0.53	0.32–0.88	0.013	0.44	0.23–0.82	0.010
NLR ≤ 3 versus NLR > 3	0.63	0.43–0.94	0.023	0.62	0.39–0.99	0.045
PLR ≤ 300 versus PLR > 300	0.51	0.33–0.81	0.004	0.63	0.38–1.02	0.062
LMR > 3 versus LMR ≤ 3	0.70	0.47–1.03	0.069	0.60	0.38–0.96	0.034

*ALC* absolute lymphocyte count, *CI* confidence interval, *HR* hazard ratio, *LMR* lymphocyte-to-monocyte ratio, *NLR* neutrophil-to-lymphocyte ratio, *OS* overall survival, *PLR* platelet-to-lymphocyte ratio, *TTF* time to treatment failure.

### Correlation between systemic immunity markers and OS

**Figure 2:**
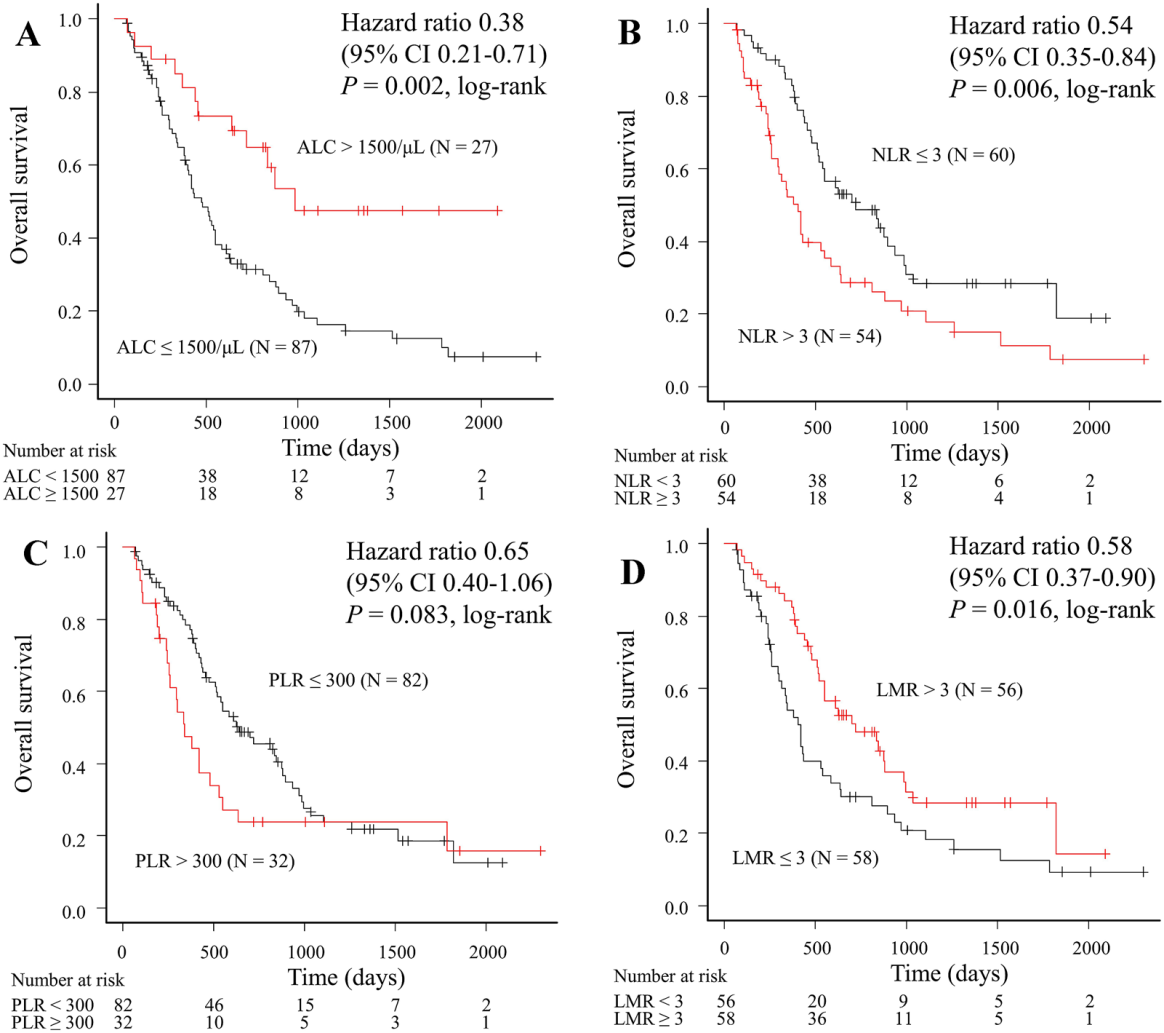
Overall survival according to baseline levels (**A**) ALC, (**B**) NLR, (**C**) PLR, and (**D**) LMR in patients treated with paclitaxel plus bevacizumab. *ALC* absolute lymphocyte count, *CI* confidence interval, *LMR* lymphocyte-to-monocyte ratio, *NLR* neutrophil-to-lymphocyte ratio, *PLR* platelet-to-lymphocyte ratio.

We compared OS relative to the systemic immunity markers (Fig. 2). The OS for patients with high ALC, low NLR, and high LMR was significantly longer compared with that for patients with low ALC (988 days vs. 475 days, log-rank *P* = 0.002; Fig. 2A), high NLR (722 days vs. 403 days, log-rank *P* = 0.006; Fig. 2B), and low LMR (722 days vs. 418 days, log-rank *P* = 0.016; Fig. 2D), respectively. Although PLR showed a favorable tendency, no significant difference was observed (639 days vs. 342 days, log-rank *P* = 0.083 for low PLR; Fig. 2C).

We performed univariable and multivariable analyses to evaluate independent predictors of PB therapy (Tables 2 and 3). Each of the four multivariable analyses identified high ALC, low NLR, and high LMR as independent predictive markers for OS (*P* = 0.010, *P* = 0.045, and *P* = 0.034, respectively). The respective results of the four multivariate Cox regression analyses are shown in Supplemental Table S3.

### Correlation between systemic immunity markers and ORR

We compared the ORR relative to the systemic immunity markers and found no significant differences in ORR between the groups: 81.5% and 65.5% for the patients with high and low ALC, respectively (*P* = 0.15); 76.7% and 61.1% for the patients with low and high NLR, respectively (*P* = 0.10); 73.2% and 59.4% for the patients with low and high PLR, respectively (*P* = 0.18); and 74.1% and 64.3% for the patients with high and low LMR, respectively (*P* = 0.31).

### Safety

Of the 114 patients, 81 (71.1%) discontinued PB therapy as a result of disease progression, 16 (14.0%) discontinued because of adverse events, 12 (10.5%) discontinued due to “other reasons,” and 5 (4.4%) continued the therapy to the data cut-off point. There were no new recorded therapy-related adverse events.

## Discussion

Our results identified that patients with HER2-negative ABC and high ALC, low NLR, and low PLR at baseline had significantly improved TTF compared with those with low ALC, high NLR, and high PLR, respectively. We also found that high ALC, low NLR, and high LMR were associated with longer OS. We demonstrated the usefulness of these systemic immunity markers as predictive markers of PB therapy for patients with HER2-negative ABC. These markers can be easily calculated from readily available blood tests routinely measured in clinical practice.

Recent studies have demonstrated that systemic immunity markers (ALC, NLR, PLR, and LMR) are associated with better outcomes for patients with various carcinomas, including breast cancer^9–17^. It has been reported that a low ALC before the initiation of systemic therapy is associated with shorter OS and PFS in advanced carcinomas, sarcomas, and lymphomas. In addition, a low baseline ALC is strongly associated with reduced disease-free survival (DFS) (hazard ratio [HR] 5.24, 95% CI 2.23–12.30, *P* < 0.001) and OS (HR 6.13, 95% CI 2.21–17.0,* P* < 0.001) for patients with breast cancer who undergo primary chemotherapy^11,14^. High ALC (≥ 1000/μL or 1500/µL) was identified as an independent predictive marker for better OS in patients with ABC receiving eribulin therapy^19,20^. In a previous meta-analysis of patients with unselected solid tumors, high NLR was associated with poor OS (HR 1.81, 95% CI 1.67–1.97, *P* < 0.001). In patients with breast cancer, high NLR (higher than the cut-off value) was significantly associated with poor OS (HR 2.56, 95% CI 1.96–3.35, *P* < 0.001) and DFS (HR 1.74, 95% CI 1.47–2.07, *P* < 0.001)^10,15^. Further, low NLR (< 3) at baseline was significantly associated with longer PFS^18^ and OS^20^ in patients treated with eribulin in ABC, thereby demonstrating that low NLR (< 3) was an independent predictive marker for eribulin therapy. It has been reported that high PLR is associated with shorter OS for various solid tumors and a meta-analysis showed that high PLR was associated with poor OS (HR 1.55, 95% CI 1.07–2.25, *P* = 0.022) and DFS (HR 1.73, 95% CI 1.30–2.30, *P* < 0.001) in patients with breast cancer^12,16^. Another systematic review and meta-analysis that included 11,197 patients from 29 studies showed that low LMR (lower than the cut-off) was associated with poor OS (HR, 1.73 95% CI 1.55–1.93, *P* < 0.001) and DFS (HR 1.56, 95% CI 1.31–1.86, *P* < 0.001) in non-hematologic solid tumors^13^. Ni et al. demonstrated that high LMR predicted a favorable response and prognosis in ABC^17^. However, the utility of PLR and LMR as predictive markers for patients with ABC remains unclear. Our results showed that high ALC (≥ 1500/µL) and low NLR (< 3) were associated with longer TTF and OS compared with low ALC and high NLR, respectively. Furthermore, we found that low PLR was associated with longer TTF and high LMR was associated with longer OS.

Our results support those of a previous study reporting that systemic immunity marker expression at the beginning of PB therapy exhibited a significant association with improved prognosis and that these markers were readily available as predictive markers of PB therapy in ABC^23^. In contrast, NLR and ALC showed no significant association with increased PFS and OS in patients with ABC treated with nab-paclitaxel or a treatment of the physician’s choice, such as taxanes^18,20^. In advanced non-small-cell lung cancer and metastatic colorectal cancer, NLR was associated with survival outcome and was useful as a predictive marker in patients treated with combination chemotherapy and bevacizumab, but not with chemotherapy alone^21,22^. Therefore, we conclude that systemic immunity markers can be useful as predictive markers by addition of bevacizumab to chemotherapy.

Recently, vascular endothelial growth factor (VEGF) has been recognized as an important mediator of immune suppression, and VEGF blockade may be effective in the antitumor immune response in addition to its direct effects on tumor vasculature. VEGF modulates the various processes of cancer immunity, including promotion of T-regulatory cells, suppression of dendritic cell maturation, stimulation of tumor-associated macrophages, and infiltration of myeloid-derived suppressor cells, leading to an immunosuppressive state^24^. Given that bevacizumab modulates this immunosuppressive state through angiogenesis inhibition, a strategy that combines bevacizumab and immune checkpoint inhibitors has been explored. Combined ipilimumab and bevacizumab therapy in patients with melanoma resulted in encouraging antitumor activity and had beneficial effects on the host antitumor immune response^25^. In patients with metastatic non-squamous non-small-cell lung cancer, the addition of atezolizumab to bevacizumab and chemotherapy significantly increased PFS and OS^26^. Therefore, it is reasonable to suggest that systemic immunity markers can predict systemic antitumor activity resulting from PB therapy in patients with ABC.

This study had several limitations. First, it was a retrospective single-center study with a small number of subjects. Given that this was a single-center study, however, the patients were treated consistently and only two (1.8%) patients were untraceable. Second, the optimal cut-off values for the systemic immunity markers are unclear. Because the cut-off values for ALC at 1500/μL and NLR at 3 have often been reported as useful in ABC^18,20,23^, we followed these parameters. Finally, which systemic immunity marker is the most effective at predicting survival remains unclear. Therefore, further prospective studies are warranted to resolve this issue. Overall, our study demonstrated the usefulness of systemic immunity markers associated with lymphocytes as predictive markers of PB therapy for patients with HER2-negative ABC. We also demonstrated that these systemic immunity markers may play an important role in selecting candidates with HER2-negative ABC for bevacizumab therapy.

## Methods

### Study population and treatment

We reviewed the medical records of 114 patients with HER2-negative ABC who underwent at least two cycles of PB therapy at the Fukuyama City Hospital (Japan) from November 2011 to December 2019. We excluded patients with missing systemic immunity marker data and patients who had not undergone at least two cycles of PB therapy. The data cut-off was May 31, 2020. We defined the subtype from the pathology reports at the time of surgery, initial biopsy, or at the time of recurrence. The subtype was based on ER and HER2 expression, and ER-positivity was defined as ER ≥ 1% positive. HER2 overexpression was defined according to the American Society of Clinical Oncology/College of American Pathologists guidelines^27^.

The treatment schedule was the same as that of the E2100 study^1^: Bevacizumab (10 mg/kg) was administered on days 1 and 15 in combination with paclitaxel (90 mg/m^2^) on days 1, 8, and 15 of each 28-day cycle. Treatment was continued until disease progression, unacceptable toxicity, or patient/physician decision. Tumor response to treatment was assessed according to the Response Evaluation Criteria in Solid Tumors (RECIST) version 1.1, and the physician determined the timing of the lesion assessment.

All procedures that involved human subjects were performed in accordance with the ethical standards of the institutional and/or national research committees and with the 1964 Helsinki Declaration and its later amendments or comparable ethical standards. This retrospective study was approved by the Fukuyama City Hospital’s review board. Informed consent was obtained in the form of an opt-out on the website from all individual participants included in the study.

### Measurements of systemic immunity markers

Neutrophil, lymphocyte, platelet, and monocyte counts were performed automatically using a Sysmex XE-2100 or XE-5000 automated hematology system (Sysmex Co., Kobe, Japan). ALC, NLR, PLR, and LMR were calculated from blood cell counts prior to administering PB therapy, and the cut-off values for these markers were set in accordance with previous studies as follows: 1500/μL for ALC, 3 for NLR, 300 for PLR, and 3 for LMR^12,13,15,20^. All patients were divided into “low” and “high” groups according to the cut-off values, respectively: low ALC (≤ 1500/μL, n = 87), high ALC (> 1500/μL, n = 27); low NLR (≤ 3, n = 60), high NLR (> 3, n = 54); low PLR (≤ 300, n = 82), high PLR (> 300, n = 32); and low LMR (≤ 3, n = 56), high LMR (> 3, n = 58). Neutrophil, lymphocyte, platelet, and monocyte counts are routinely measured in clinical practice during treatment and the systemic immunity markers are easily calculated from these blood cell counts. Therefore, these markers can be measured and analyzed easily and inexpensively without additional equipment, software, or personnel specialized in analyzing the results.

### Statistical analysis

The Wilcoxon rank sum test was used to compare continuous variables (such as median age) and Fisher’s exact test was used to compare the proportions of categorical variables (such as metastasis type) between groups. The distribution of TTF and OS was estimated by the Kaplan–Meier method. We performed a univariate Cox regression analysis to determine the association between baseline patient characteristics and TTF and OS. To evaluate the association between each systemic immunity marker (ALC, NLR, PLR, and LMR) and TTF or OS, we conducted a multivariate Cox regression analysis. We considered the baseline patient characteristics with *P* < 0.20 in the univariate Cox regression analysis as confounders and included them in the multivariate analysis. Since the systemic immunity markers (ALC, NLR, PLR, and LMR) were correlated with each other, we did not include these four markers simultaneously in the multivariate analysis. These markers were included independently in each multivariate analysis of TTF and OS. Finally, we performed this analysis eight times. A *P* value < 0.05 was considered statistically significant, and all statistical analyses were performed with EZR software (Saitama Medical Center, Jichi Medical University, Saitama, Japan), a graphical user interface for R (The R Foundation for Statistical Computing, Vienna, Austria)^28^.

We defined TTF as the time from the administration of PB therapy to the discontinuation of treatment for any reason, including disease progression, treatment toxicity, patient/physician decision, and death from any cause. OS was defined as the time from the administration of PB therapy to the date of death from any cause. ORR was defined as the percentage of patients who achieved a complete or partial response according to the RECIST criteria.

## Supplementary Information

Supplementary information.

## Data Availability

The datasets generated during and/or analyzed during the current study are available from the corresponding author upon reasonable request.
