# Supplementary material for: Systemic immunity markers associated with lymphocytes predict the survival benefit from paclitaxel plus bevacizumab in HER2 negative advanced breast cancer
Source: Sci Rep. 2021 Mar 18;11:6328. doi: 10.1038/s41598-021-85948-2 (PMC7973794; doi:10.1038/s41598-021-85948-2)
Supplement: Supplementary file 1 — Supplementary information. [file 41598_2021_85948_MOESM1_ESM.doc]

**Systemic immunity markers associated with lymphocytes predict the survival benefit from paclitaxel plus bevacizumab in HER2 negative advanced breast cancer**

Shogo Nakamoto^1^, Masahiko Ikeda^1^, Shinichiro Kubo^1^, Mari Yamamoto^1^, Tetsumasa Yamashita^1^, Akifumi Notsu ^2^

^1^ Division of Breast and Thyroid Gland Surgery, Fukuyama City Hospital, Hiroshima, Japan

^2^ Division of Clinical Research Center, Shizuoka Cancer Center, Shizuoka, Japan

**Corresponding author:** Shogo Nakamoto, Department of Breast and Thyroid Gland Surgery, Fukuyama City Hospital, 5-23-1 Zao, Fukuyama city, Hiroshima pref., Japan. Tel.: +81-84-941-5151, Fax: +81-84-941-5159, e-mail: [nakamoto0246@fchp.jp](mailto:nakamoto0246@fchp.jp)

**ORCiD IDs**

SN: 0000-0001-8043-8608

MI: not available

SK: not available

MY: not available

TY: not available

AN: not available

**Online Resource Supplemental Table S1.** Patient characteristics: Systemic immunity markers at the beginning of paclitaxel plus bevacizumab therapy

| Variables | ALC | |  | NLR | |  | PLR | |  | LMR | |  |
| --- | --- | --- | --- | --- | --- | --- | --- | --- | --- | --- | --- | --- |
|  | Low (n = 87) | High (n = 27) | *P* | Low (n = 60) | High (n = 54) | *P* | Low (n = 82) | High (n = 32) | *P* | Low (n = 56) | High (n = 58) | *P* |
| Age, years, median (range) | 62.0 (36–89) | 62.0 (32–76) | 0.594 | 62.5 (32–83) | 61.0 (36–89) | 0.742 | 63.0 (32–89) | 61.0 (37–86) | 0.416 | 61.0 (36–89) | 64.0 (32–83) | 0.331 |
| ER status, N (%) |  |  |  |  |  |  |  |  |  |  |  |  |
| Positive | 63 (72.4) | 19 (70.4) | 0.811 | 43 (71.7) | 39 (72.2) | 1.000 | 58 (70.7) | 24 (75.0) | 0.817 | 39 (69.6) | 43 (74.1) | 0.678 |
| Negative | 24 (27.6) | 8 (29.6) |  | 17 (28.3) | 15 (27.8) |  | 24 (29.3) | 8 (25.0) |  | 17 (30.4) | 15 (25.9) |  |
| Diagnosis, N (%) |  |  |  |  |  |  |  |  |  |  |  |  |
| Advanced | 33 (37.9) | 11 (40.7) | 0.824 | 24 (40.0) | 20 (37.0) | 0.848 | 33 (40.2) | 11 (34.4) | 0.670 | 21 (37.5) | 23 (39.7) | 0.849 |
| Recurrence | 54 (62.1) | 16 (59.3) |  | 36 (60.0) | 34 (63.0) |  | 49 (59.8) | 21 (65.6) |  | 35 (62.5) | 35 (60.3) |  |
| Metastatic sites, N (%) |  |  |  |  |  |  |  |  |  |  |  |  |
| CNS | 8 (9.2) | 2 (7.4) | 1.000 | 3 (5.0) | 7 (13.0) | 0.188 | 4 (4.9) | 6 (18.8) | 0.028 | 7 (12.5) | 3 (5.2) | 0.200 |
| Bone | 51 (58.6) | 10 (37.0) | 0.076 | 24 (40.0) | 37 (68.5) | 0.003 | 42 (51.2) | 19 (59.4) | 0.532 | 34 (60.7) | 27 (46.6) | 0.139 |
| Lung | 39 (44.8) | 16 (59.3) | 0.270 | 34 (56.7) | 21 (38.9) | 0.064 | 38 (46.3) | 17 (53.1) | 0.538 | 21 (37.5) | 34 (58.6) | 0.026 |
| Pleura and/or lymphangiopathy | 38 (43.7) | 8 (29.6) | 0.260 | 23 (38.3) | 23 (42.6) | 0.704 | 28 (34.1) | 18 (56.3) | 0.036 | 25 (44.6) | 21 (36.2) | 0.446 |
| Lymph node | 68 (78.2) | 22 (81.5) | 0.794 | 50 (83.3) | 40 (74.1) | 0.256 | 66 (80.5) | 24 (75.0) | 0.610 | 43 (76.8) | 47 (81.0) | 0.649 |
| Liver | 46 (52.9) | 7 (25.9) | 0.016 | 22 (36.7) | 31 (57.4) | 0.038 | 38 (46.3) | 15 (46.9) | 1.000 | 31 (55.4) | 22 (37.9) | 0.091 |
| Soft tissue | 53 (60.9) | 20 (74.1) | 0.256 | 42 (70.0) | 31 (57.4) | 0.177 | 55 (67.1) | 18 (56.3) | 0.287 | 35 (62.5) | 38 (65.5) | 0.846 |
| Type of metastases, N (%) |  |  |  |  |  |  |  |  |  |  |  |  |
| Visceral | 73 (83.9) | 20 (74.1) | 0.264 | 46 (76.7) | 47 (87.0) | 0.226 | 64 (78.0) | 29 (90.6) | 0.178 | 47 (83.9) | 46 (79.3) | 0.631 |
| Non-visceral | 14 (16.1) | 7 (25.9) |  | 14 (23.3) | 7 (13.0) |  | 18 (22.0) | 3 (9.4) |  | 9 (16.1) | 12 (20.7) |  |
| Number of metastatic sites, median (range) | 4 (1–8) | 3 (1–6) | 0.337 | 3 (1–8) | 4 (1–8) | 0.340 | 3 (1–8) | 4 (1–7) | 0.068 | 4 (1–8) | 3 (1–8) | 0.588 |
| Number of metastatic sites, N (%) |  |  |  |  |  |  |  |  |  |  |  |  |
| ≥3 | 70 (80.5) | 23 (85.2) | 0.778 | 50 (83.3) | 43 (79.6) | 0.637 | 65 (79.3) | 28 (87.5) | 0.423 | 45 (80.4) | 28 (48.3) | 0.812 |
| <3 | 17 (19.5) | 4 (14.8) |  | 10 (16.7) | 11 (20.4) |  | 17 (20.7) | 4 (12.5) |  | 11 (19.6) | 10 (17.2) |  |
| Prior (neo) adjuvant chemotherapy*, N (%) |  |  |  |  |  |  |  |  |  |  |  |  |
| Yes | 31 (35.6) | 6 (22.2) | 0.243 | 19 (31.7) | 18 (33.3) | 1.000 | 24 (29.3) | 13 (40.6) | 0.271 | 19 (33.9) | 18 (31.0) | 0.842 |
| No | 56 (64.4) | 21 (77.8) |  | 41 (68.3) | 36 (66.7) |  | 58 (70.7) | 19 (59.4) |  | 37 (66.1) | 40 (69.0) |  |
| Disease-free interval, N (%) |  |  |  |  |  |  |  |  |  |  |  |  |
| <24 months | 51 (58.6) | 16 (59.3) | 1.000 | 35 (58.3) | 32 (59.3) | 1.000 | 48 (58.5) | 19 (59.4) | 1.000 | 36 (64.3) | 31 (53.4) | 0.259 |
| ≥24 months | 36 (41.4) | 11 (40.7) |  | 25 (41.7) | 22 (40.7) |  | 34 (41.5) | 13 (40.6) |  | 20 (35.7) | 27 (46.6) |  |
| Number of previous chemotherapies, N (%) |  |  |  |  |  |  |  |  |  |  |  |  |
| 0–1 | 63 (72.4) | 26 (96.3) | 0.007 | 51 (85.0) | 38 (70.4) | 0.072 | 66 (80.5) | 23 (71.9) | 0.324 | 41 (73.2) | 48 (82.8) | 0.261 |
| ≥2 | 24 (27.6) | 1 (3.7) |  | 9 (15.0) | 16 (29.6) |  | 16 (19.5) | 9 (28.1) |  | 15 (26.8) | 10 (17.2) |  |

Abbreviations: ALC, absolute lymphocyte count; CNS, central nervous system; ER, estrogen receptor; LMR, lymphocyte-to-monocyte ratio; NLR, neutrophil-to-lymphocyte ratio; PLR, platelet-to-lymphocyte ratio.

* Chemotherapy included anthracycline and/or taxane**Online Resource Supplemental Table S2.** Multivariable analysis of time to treatment failure (Cox hazard model)

|  | ALC | |  | NLR | |  | PLR | |  | LMR | |  |
| --- | --- | --- | --- | --- | --- | --- | --- | --- | --- | --- | --- | --- |
|  | HR | 95% CI | *P* | HR | 95% CI | *P* | HR | 95% CI | *P* | HR | 95% CI | *P* |
| Age (≥65 years vs. <65 years) | – | – | – | – | – | – | – | – | – | – | – | – |
| ER (negative vs. positive) | – | – | – | – | – | – | – | – | – | – | – | – |
| Diagnosis (recurrence vs. advanced) | 1.28 | 0.75–2.17 | 0.370 | 1.06 | 0.63–1.78 | 0.818 | 1.16 | 0.69–1.97 | 0.573 | 1.08 | 0.65–1.82 | 0.762 |
| Metastatic sites (yes vs. no) | – | – | – | – | – | – | – | – | – | – | – | – |
| CNS | – | – | – | – | – | – | – | – | – | – | – | – |
| Bone | – | – | – | – | – | – | – | – | – | – | – | – |
| Lung | 0.62 | 0.41–0.94 | 0.026 | 0.64 | 0.42–0.98 | 0.041 | 0.65 | 0.43–1.00 | 0.050 | 0.66 | 0.43–1.01 | 0.056 |
| Pleura and/or lymphangiopathy | – | – | – | – | – | – | – | – | – | – | – | – |
| Lymph node | – | – | – | – | – | – | – | – | – | – | – | – |
| Liver | 1.18 | 0.78–1.78 | 0.433 | 1.13 | 0.74–1.71 | 0.575 | 1.16 | 0.66–2.05 | 0.180 | 1.18 | 0.78–1.78 | 0.431 |
| Soft tissue | 1.19 | 0.68–2.08 | 0.550 | 1.10 | 0.63–1.93 | 0.735 | 1.16 | 0.66–2.01 | 0.612 | 1.06 | 0.61–1.85 | 0.837 |
| Visceral metastasis (yes vs. no) | – | – | – | – | – | – | – | – | – | – | – | – |
| Number of metastatic sites (≥3 vs. <3) | 0.91 | 0.52–1.58 | 0.740 | 0.90 | 0.52–1.57 | 0.711 | 0.81 | 0.46–1.42 | 0.464 | 0.88 | 0.51–1.53 | 0.654 |
| Prior (neo) adjuvant chemotherapy* (yes vs. no) | 1.24 | 0.73–2.10 | 0.423 | 1.42 | 0.84–2.40 | 0.191 | 1.27 | 0.75–2.14 | 0.374 | 1.41 | 0.83–2.39 | 0.198 |
| Disease-free interval (<24 months vs. ≥24 months) | – | – | – | – | – | – | – | – | – | – | – | – |
| Number of previous chemotherapies (<2 vs. ≥2) | – | – | – | – | – | – | – | – | – | – | – | – |
| Marker of systemic immunity | – | – | – | – | – | – | – | – | – | – | – | – |
| ALC > 1500/μL vs. ALC ≤ 1500/μL | 0.53 | 0.32–0.88 | 0.013 | – | – | – | – | – | – | – | – | – |
| NLR ≤ 3 vs. NLR > 3 | – | – | – | 0.63 | 0.43–0.94 | 0.023 | – | – | – | – | – | – |
| PLR ≤ 300 vs. PLR > 300 | – | – | – | – | – | – | 0.51 | 0.33–0.81 | 0.004 | – | – | – |
| LMR > 3 vs. LMR ≤ 3 | – | – | – | – | – | – | – | – | – | 0.70 | 0.47–1.03 | 0.069 |

Abbreviations: ALC, absolute lymphocyte count; CI, confidence interval; CNS, central nervous system; ER, estrogen receptor; HR, hazard ratio; LMR, lymphocyte-to-monocyte ratio; NLR, neutrophil-to-lymphocyte ratio; PLR, platelet-to-lymphocyte ratio.

* Chemotherapy included anthracycline and/or taxane

**Online Resource Supplemental Table S3.** Multivariable analysis of overall survival (Cox hazard model)

|  | ALC | |  | NLR | |  | PLR | |  | LMR | |  |
| --- | --- | --- | --- | --- | --- | --- | --- | --- | --- | --- | --- | --- |
|  | HR | 95% CI | *P* | HR | 95% CI | *P* | HR | 95% CI | *P* | HR | 95% CI | *P* |
| Age (≥65 years vs. <65 years) | – | – | – | – | – | – | – | – | – | – | – | – |
| ER (negative vs. positive) | – | – | – | – | – | – | – | – | – | – | – | – |
| Diagnosis (recurrence vs. advanced) | 1.28 | 0.79–2.06 | 0.315 | 1.22 | 0.75–1.96 | 0.422 | 1.18 | 0.73–1.89 | 0.505 | 1.27 | 0.78–2.06 | 0.332 |
| Metastatic sites (yes vs. no) | – | – | – | – | – | – | – | – | – | – | – | – |
| CNS | – | – | – | – | – | – | – | – | – | – | – | – |
| Bone | – | – | – | – | – | – | – | – | – | – | – | – |
| Lung | 0.73 | 0.46–1.15 | 0.175 | 0.76 | 0.47–1.23 | 0.261 | 0.71 | 0.45–1.14 | 0.155 | 0.79 | 0.49–1.29 | 0.347 |
| Pleura and/or lymphangiopathy | – | – | – | – | – | – | – | – | – | – | – | – |
| Lymph node | – | – | – | – | – | – | – | – | – | – | – | – |
| Liver | 1.14 | 0.71–1.83 | 0.583 | 1.10 | 0.68–1.79 | 0.697 | 1.17 | 0.72–1.90 | 0.519 | 1.13 | 0.70–1.84 | 0.620 |
| Soft tissue | – | – | – | – | – | – | – | – | – | – | – | – |
| Visceral metastasis (yes vs. no) | – | – | – | – | – | – | – | – | – | – | – | – |
| Number of metastatic sites (≥3 vs. <3) | – | – | – | – | – | – | – | – | – | – | – | – |
| Prior (neo) adjuvant chemotherapy* (yes vs. no) | – | – | – | – | – | – | – | – | – | – | – | – |
| Disease-free interval (<24 months vs. ≥24 months) | – | – | – | – | – | – | – | – | – | – | – | – |
| Number of previous chemotherapies (<2 vs. ≥2) | 0.60 | 0.36–1.02 | 0.057 | 0.54 | 0.32–0.90 | 0.019 | 0.48 | 0.29–0.81 | 0.006 | 0.50 | 0.30–0.84 | 0.009 |
| Marker of systemic immunity | – | – | – | – | – | – | – | – | – | – | – | – |
| ALC > 1500/μL vs. ALC ≤ 1500/μL | 0.44 | 0.23–0.82 | 0.010 | – | – | – | – | – | – | – | – | – |
| NLR ≤ 3 vs. NLR > 3 | – | – | – | 0.62 | 0.39–0.99 | 0.045 | – | – | – | – | – | – |
| PLR ≤ 300 vs. PLR > 300 | – | – | – | – | – | – | 0.63 | 0.38–1.02 | 0.062 | – | – | – |
| LMR > 3 vs. LMR ≤ 3 | – | – | – | – | – | – | – | – | – | 0.60 | 0.38–0.96 | 0.034 |

Abbreviations: ALC, absolute lymphocyte count; CI, confidence interval; CNS, central nervous system; ER, estrogen receptor; HR, hazard ratio; LMR, lymphocyte-to-monocyte ratio; NLR, neutrophil-to-lymphocyte ratio; PLR, platelet-to-lymphocyte ratio.

* Chemotherapy included anthracycline and/or taxane
